# Mouse dead end 1-β interacts with c-Jun and stimulates activator protein 1 transactivation

**DOI:** 10.3892/mmr.2014.2950

**Published:** 2014-11-14

**Authors:** YONG ZHANG, YAN-LIN SU, LE-SAI LI, ZHI YANG, SI CHEN, JIE XIONG, XIAO-HUA FU, XIAO-NING PENG

**Affiliations:** 1 Department of Internal Medicine, College of Medicine, Hunan Normal University, Changsha, Hunan 410013, P.R. China; 2 Department of Gynecologic Oncology, Cancer Hospital of Xiangya School of Medicine of Central South University, Changsha, Hunan 410013, P.R. China

**Keywords:** dead end 1-β, c-Jun, activator protein-1, yeast two-hybrid, interaction, immunoprecipitation, glutathione 3-transferase pull-down

## Abstract

Dead end 1 (DND1), important for maintaining the viability of primordial germ cells, is the first protein containing an RNA recognition motif that has been directly implicated as a heritable cause of spontaneous tumorigenesis. In the present study, c-Jun was identified through yeast two-hybrid screening of a 10.5-day old mouse embryo cDNA library as one of the proteins which interact with DND1-β. The interaction between DND1-β and c-Jun was demonstrated to occur by glutathione S-transferase pull-down and co-immunoprecipitation. Using confocal microscopy, DND1-β was found to be specifically expressed in GC-1 spermatogonia cells, mainly in the nuclei. When transfected into GC-1 cells, DND1-β and c-Jun were demonstrated to be co-localized principally in the nuclei. Furthermore, in a dual luciferase reporter assay, the transcriptional activity of activator protein 1 was demonstrated to be significantly increased by co-transfection with DND1-β and c-Jun plasmids in GC-1 cells. The identification and confirmation of an additional protein interacting with DND1-β facilitates the investigation of the functions and molecular mechanisms of DND1.

## Introduction

The dead end (Dnd1) gene was first characterized as a gene required for germ-cell viability in a large-scale screening of zebrafish (1–3). The mouse Dnd1 gene encodes two protein isoforms: DND1-isoform α (DND1-α), 352 amino acids in length, and DND1-isoform β (DND1-β), 340 amino acids in length. DND1 is found in the fetal testis during the critical period when testicular germ cell tumors (TGCTs) are hypothesized to develop in mice (3). In a previous study, DND1 was demonstrated to be critical for germ cell development (3–5) and embryonic viability (6) in mice. The likely functional inactivation of the Dnd1 gene in mice results in severe germ cell depletion and development of TGCT.

DND1 is a highly conserved RNA-binding protein important for maintaining the viability of primordial germ cells (PGCs) in vertebrates, including zebrafish, frogs and mice (1,3,7). Knockdown of Dnd1 in zebrafish resulted in loss of PGC motile behavior and subsequent PGC death (1). Dnd1 mutations have been directly implicated as a heritable cause of spontaneous tumorigenesis in mice. In 129 inbred mice, a point mutation in Dnd1 introduced a stop codon, which resulted in truncated Dnd1, which may give rise to PGC deficiency, testicular germ cell tumor development and partial embryonic lethality (3). Human Dnd1 maps to chromosome 5q31.3. Chromosomal deletions in human 5q have been identified in germ-cell tumor tissues and germ-cell tumor cell lines, and Dnd1 mutations have also been found in TGCTs (8,9).

The mechanism by which the DND1 regulates cell migration, germ cell survival and TGCT development has yet to be elucidated. Thus far, studies have found that DND1-α is expressed in early embryos and in the testis following birth, whereas DND1-β is expressed in the germ cells of adult testis. DND1-α aids in maintaining PGC viability and the loss of DND1-α results in transformation of the PGC cells into embryonal carcinoma cells. However, the role and mechanism of DND1-β in adult germ cells remains elusive (1,6). In the present study, proteins that interact with DND1-β were identified using a yeast two-hybrid interaction strategy.

## Materials and methods

### Plasmid construction

The pcDNA3.1+ vector was purchased from Invitrogen Life Technologies (Carlsbad, CA, USA) in order to construct the pcDNA3.1-Dnd1-β expression plasmid. The Dnd1-β gene (GenBank ID: BC034897.1) was amplified by reverse transcription polymerase chain reaction (RT-PCR) from the mRNA of splenocytes derived from C57BL/6J mice, obtained from Dr. Matin Angabin ( Texas University MD Anderson Cancer Center, Houston, TX, USA). The primers were as follows: Forward 5′-CAGATCTGATGGTCTCCCTCCCATCCCAA-3′ and reverse 5′-GGAATTCCTCACTGCTTAACCATAGTACC-3′ for Dnd1-β. Dnd1-β cDNA was purified by Shanghai Biological Engineering Company (Shanghai, China). For yeast two-hybrid screening, full-length Dnd1-β cDNA was ligated in-frame with the GAL4 DNA-binding domain of the pDBLeu vector, resulting in pDBLeu/Dnd1-β. For the immunoprecipitation assay, full-length Dnd1-β cDNA was cloned into the mammalian expression plasmid p cytomegalovirus (CMV)-Myc vector (Clontech Laboratories, Mountain View, CA, USA), forming the Myc-tagged Dnd1-β expression vector, pCMV-Myc-Dnd1-β. Full-length c-Jun cDNA was inserted into a pCMV-human influenza haemagluttinin (HA) vector (Clontech Laboratories), forming the HA-tagged c-Jun expression vector, pCMV-HA-c-Jun. For the co-localization assay, full-length Dnd1-β cDNA was cloned into a p enhanced green fluorescent protein (EGFP)-c3 vector, forming the pEGFP-c3-Dnd1-β vector, and full-length c-Jun cDNA was inserted into a pm red fluorescent protein (RFP) vector Clontech Laboratories), forming the pmRFP-c-Jun vector. The pGEX-4T-2 vector (GE Healthcare Life Sciences Corp, Shanghai, China) was used to construct vectors expressing glutathione S-transferase (GST)-DND1-β fusion proteins. The cDNA fragment encoding full-length Dnd1-β was cloned in-frame with respect to GST into the pGEX-4T-2 vector. The pQE-N3 Plasmid (Qiagen, Hilden, Germany) was used to generate vectors expressing polyhistidine (His)-tagged c-Jun fusion proteins. AP1-luc (Stratagene, La Jolla, CA, USA) is a reporter plasmid encoding the firefly luciferase gene driven by several copies of an AP-1 enhancer element. The pCMV-LacZ vector was constructed by fusing the LacZ gene to pCMV-Myc (Qiagen, Hilden, Germany).

### Cell culture and transient transfection

The GC-1 cell line (CRL-2053; American Type Culture Collection, Manassas, VA, USA). was cultured in Dulbecco’s modified Eagle’s medium (DMEM; Sigma-Aldrich, St. Louis, MO, USA) supplemented with 10% fetal bovine serum (Invitrogen Life Technologies), penicillin (100 U/ml) and streptomycin (100 mg/ml) in a 5% CO_2_ atmosphere at 37°C. The GC-1 cells were plated in collagen-coated dishes and were harvested at 100% confluency for co-immunoprecipitation analysis and immunofluorescence. The GC-1 cells were transiently transfected with plasmid constructs at 70% confluence using Lipofectamine 2000 (Invitrogen Life Technologies) according to the manufacturer’s instructions. After 48 h transfection time, the cells were harvested for the co-immunoprecipitation assay.

### Yeast two-hybrid analysis

The ProQuest™ yeast two-hybrid system was obtained from Invitrogen Life Technologies. A 10.5-day old mouse embryo cDNA library (Invitrogen Life Technologies) cloned in-frame with the GAL4 activation domain in the pPC86 vector (Invitrogen Life Technologies) was used to screen for Dnd1-β-interacting clones. The MaV203 yeast strain (Gibco-BRL, Carlsbad, CA, USA) was transformed with pDBLeu-Dnd1-β and analyzed for basal expression activity, as described in the manufacturer’s instructions. The bait-containing MaV203 cells were subsequently transformed with the 10.5-day old mouse embryo cDNA library and transformants were selected by growing the cells in medium lacking leucine, tryptophan, uracil and histidine (SD-leu-, Trp-, Ura-, His-) supplemented with 30 mM 3-amino-1,2,4-triazole (3-AT; Invitrogen Life Technologies). False positive clones were eliminated by retransforming the prey DNA to the original bait strain and positive clones were further verified using an X-gal filter assay. The plasmids from positive clones were sequenced and characterized as previously described (10).

### GST pull-down assay

The GST pull-down assay was performed as previously described (11). The BL21 (DE3) cells were transformed with plasmids encoding GST, pGEX-4T-2/Dnd1-β and pQE3/c-Jun fusion proteins. Overnight cultures were induced for 4 h with 0.1 mM isopropyl-β-d-thiogalactopyranoside (GE Healthcare Life Sciences Corp), subsequent to which the bacteria were harvested, resuspended in phosphate-buffered saline (PBS) containing 1% (vol/vol) Triton X-100 and lysozyme (0.1 mg/ml), and lysed by sonication. The bacterial extracts were clarified and mixed with 50% (vol/vol) glutathione-Sepharose 4B ((GE Healthcare Life Sciences Corp) slurry in PBS (50 μl per 1 ml of lysates). After 15 min on ice, the beads were pelleted, washed and resuspended in 0.4 ml portions of either cytoplasmic supernatant from c-jun-infected cell lysates containing *in vitro*-translated c-jun. After 30 min, the beads were pelleted and washed. Complexes of GST-DND1-β bound to c-jun were eluted with a small volume of 10 mM glutathione in 50 mM Tris (pH 8.0) and analyzed by SDS-PAGE. [^35^S]methionine-labeled c-Jun from the *in vitro* translation reactions was detected by autoradiography (x-ray film) of the dried gel, while unlabeled c-Jun from the lysates of infected cells was detected by Western blot analysis with mouse monoclonal anti-c-Jun antibody (Abcam, Cambridge, MA, USA).

### Co-immunoprecipitation

GC-1 cells were co-transfected with pCMV-Myc-Dnd1-β and pCMV-HA-c-jun. At 24 h after transfection, the GC-1 cells were washed with PBS and homogenized in co-immunoprecipitation lysis buffer [20 mM hydroxyethyl piperazineethanesulfonic acid, pH 7.4, 125 mM NaCl, 1% Triton X-100, 10 mM ethylene glycol tetraacetic acid (EGTA), 2 mM Na_3_VO_4_, 50 mM NaF, 20 mM ZnCl_2_, 10 mM sodium pyrophosphate, 1 mM dithiothreitol and 1 mM phenylmethylsulfonyl fluoride]. Subsequently, 1X Complete Protease Inhibitor mixture (Sigma-Aldrich, Shanghai, China) was added prior to use. The supernatant fractions were collected and incubated with mouse monoclonal anti-Myc antibody or rabbit polyclonal anti-HA antibody (Abcam; 1:200) and GammaBind Plus-Sepharose beads (Amersham Pharmacia Biotech) with agitation overnight at 4°C as previously described (12). Co-precipitated proteins were subjected to electrophoresis by 13% SDS-PAGE, and then analyzed by western blotting using rabbit polyclonal anti-HA antibody, monoclonal anti-Myc antibody or rabbit polyclonal anti-Dnd1-β antibody (Santa Cruz Biotechnology, Inc., Santa Cruz, CA, USA).

### Western blot analysis

Proteins were analyzed by SDS-PAGE and then transferred to a nitrocellulose membrane. After >4 h blocking in 5% milk with TBST buffer (20 mM Tris-HCl, pH 7.6, 137 mM NaCl and 0.5% Tween 20), the blots were incubated with appropriate primary antibodies at 4°C overnight. Subsequent to washing with TBST for 30 min, the membranes were incubated with 1:2,000 corresponding alkaline phosphatase-conjugated secondary antibodies for 2 h. Following another wash with TBST for 30 min, immunoreactivity was visualized by enhanced chemiluminescence (ECL; Pierce Biotechnology, Inc., Rockford, IL, USA). The images were captured on X-ray film (Kodak, Rochester, NY, USA) and protein quantification was determined using Gel Pro Analyzer 4 image analysis software (Media Cybernetics, Inc., Rockville, MD, USA).

### Confocal microscopy

GC-1 cells were transiently co-transfected with pEGFP-c3-Dnd1-β and pmRFP-c-Jun on glass slide chambers. Adherent cells were washed three times with PBS containing 2 mM EGTA and then fixed for 30 min in 3% paraformaldehyde in PBS with 2 mM EGTA. Subsequent to two rinses with PBS, the cells were permeabilized for 2 min in 0.05% Triton X-100, followed by two additional rinses with PBS. The chamber slides were observed with a Zeiss LSM 510 confocal laser scanning microscope (LSM; Carl Zeiss AG, Oberkochen, Germany) with a 100x oil (1.4-numerical aperture) immersion objective. Briefly, GFP-DND1-β and RFP-c-Jun were observed in GFP and RFP channels, respectively. The obtained images were merged to compare the two signal patterns and the overlapping areas of the images were measured using the overlay tool of LSM Image Browser software (Ver. 4.2.0.121; Carl Zeiss AG). The images were converted to 8-bit gray scale using Image J software (Ver. 1.43b) and the analysis was performed with the co-localization-finder plug-in. Merged compartments with >95% overlap were determined to be co-localized.

### Dual-luciferase reporter assay

At 24 h prior to transfection, 1×10^5^ cells/well were plated in a 24-well plate at a density of 60–80% confluence and transfected in the same medium using DMEM. The luciferase reporter PCMV-HA-DND1-β containing full-length Dnd1-β promoter and AP-1 luciferase reporter pAP-1-luc containing seven AP-1 binding sites were used for Dnd1-β promoter and AP-1 transcriptional activity assays, respectively, in cells under various conditions. The pRL-TK vector (Promega Corporation, Madison, WI, USA) served as an internal control. In each transfection, the cells were transfected with various combinations of a luciferase reporter plasmid and the desired expression vectors. The quantity of each expression vector was identical unless specified otherwise. Total DNA quantity was maintained at a constant level with pCMV or the relevant empty vectors. Transient cell transfections with AP1-Luc, pCMV-LacZ and the indicated expression vectors were performed using Lipofectamine 2000. At 24 h after transfection, the cells were lysed and dual luciferase assays were performed (Promega Corporation). Luciferase activity was normalized to renilla luciferase activity. The results are presented as the fold induction, which is the relative luciferase activity of the treated cells over that of control cells. All transfection experiments were conducted in triplicate wells and repeated separately at least three times.

### Statistical analysis

The significance of the differences among values was determined by one-way analysis of variance followed by Dunnett’s multiple comparison tests. An unpaired Student’s t-test was also applied. P<0.05 was considered to indicate a statistically significant difference. All data are presented as mean ± standard error of the mean.

## Results

### Identification of c-Jun as a DND1-β-binding protein using yeast two-hybrid assay

**Figure 1:**
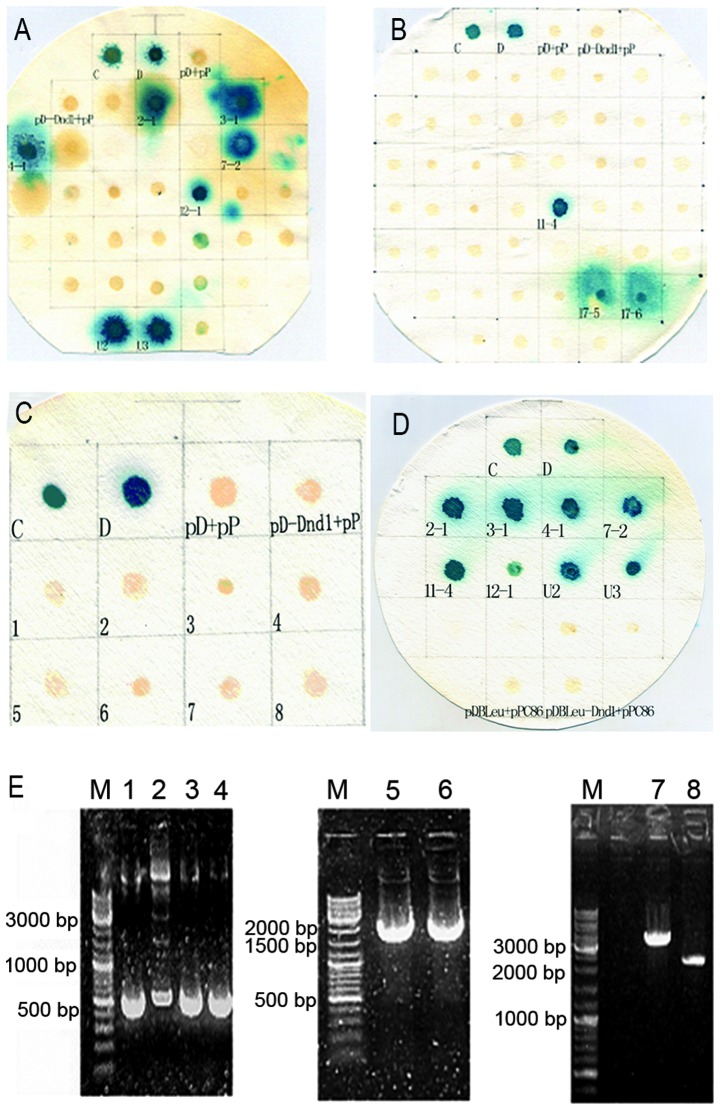
(A and B) Positive colonies from 10.5 day-old mouse embryo cDNA library screening. (C and D) Positive control colonies pD+pP, MaV203 yeast cell colonies transformed with bait vector pDBLeu and target vector pPC86; pD-Dnd1-β+pP, MaV203 cells transformed with bait plasmid pDBLeu-Dnd1-β and target vector pPC86, serving as negative controls; (A) 2-1, 3-1, 4-1, 7-2, 12-1, U2, U3 and (B) 11-4, positive colonies obtained from MaV203-pDBLeu-Dnd1-β transformed with mouse embryo cDNA library and subsequently screening of the transformants. (C and D) Assessment of self-activation of the eight positive library plasmids and confirmation of the specificity of the interaction of the putative eight targets with pDBLeu-Dnd1-β. (C) Analysis of self-activation of the eight positive library plasmids: C and D, positive control colonies; pD+pP and pD-Dnd1-β+pP, negative controls as previously described; 1–8, MaV203 cells cotransformed with the eight positive library plasmids with pDBLeu, respectively. (D) Verification of the interaction of the eight positive targets with pDBLeu-Dnd1-β in yeast: C and D, positive control colonies; pD+pP and pD-Dnd1-β+pP, negative controls; 2-1, 3-1, 4-1, 7-2, 11-4, 12-1, U2 and U3, positive colonies. (E) Identification of the positive colonies by polymerase chain reaction. Lane M, DNA marker (DL2000); Lane 1, pPC86-2-1; Lane 2, pPC86-3-1; Lane 3, pPC86-4-1; Lane 4, pPC86-7-2; Lane 5, pPC86-11-4; Lane 6, pPC86-12-1; Lane 7, pPC86-U3-1; Lane 8, pPC86-U3-2. Dnd, dead end.

To identify the proteins that associate with DND1-β, a yeast two-hybrid screening was performed with a 10.5-day old mouse embryo cDNA library and the full-length Dnd1-β as the bait. The transactivational activity of the GAL4-DND1-β fusion protein in yeast was inhibited by 30 mM 3-AT. A total of 172 positive clones were obtained in the first round of screening the mouse embryo cDNA library, 10 positive clones were obtained in the second round and 8 positive clones were confirmed as positive in the third round (Fig. 1). These eight sequences were sequenced and compared with the sequences in GenBank; four of the corresponding proteins were found to interact with DND1-β. Through bioinformatic analysis, these four proteins were identified as c-Jun, actinin alpha 1, nuclear receptor binding protein 1 and chloride channels 2. As the association between c-Jun expression and TGCT has been widely investigated, c-jun was selected for further experiments.

### DND1-β interacts with c-Jun in GST pull-down assay

**Figure 2:**
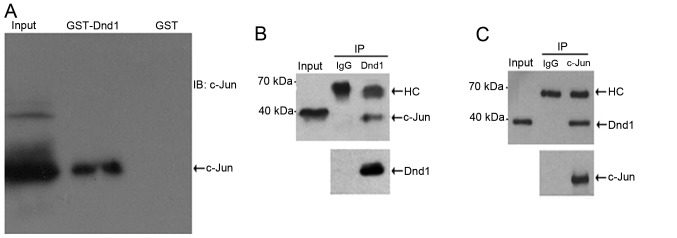
DND1-β interacted with full-length c-Jun (A) *in vitro* and (B and C) *in vivo*. (A) GST pull-down. GST-DND1-β: GST-DND1-β incubated with purified His-c-Jun. Input, purified His-c-Jun; GST, GST incubated with purified His-c-Jun. (B and C) Co-immunoprecipitation assay. Transfected cell lysates were prepared and immunoprecipitated with (B) monoclonal anti-DND1-β antibodies and (C) anti-c-Jun antibodies followed by immunoblotting with an anti-c-Jun and anti-DND1-β antibody, respectively. DND, dead end; GST, glutathione S-transferase His, histidine; IgG, immunoglobulin G; IP, immunoprecipitation; IB, immunoblot; HC, heavy chain.

The DND1-β-c-Jun interaction is possibly indirect, as other protein factors in the whole cell extract may mediate the interaction, for example, by acting as ‘bridging’ factors. To determine whether DND1-β and c-Jun interact in mammalian cells, GST pull-down assays were performed using lysates from GC-1 cells. GST, GST fusion proteins and His fusion proteins were expressed and purified. The results revealed that c-Jun was pulled down by GST-fused DND1-β but not by GST alone, indicating that DND1-β and c-Jun specifically and directly interact *in vitro* (Fig. 2A).

### DND1-β and c-Jun are co-immunoprecipitated in GC-1 cells

To determine whether endogenous DND1-β interacts with c-Jun in mammalian cells, a co-immunoprecipitation experiment was conducted in GC-1 cells with anti-DND1-β, anti-c-Jun and non-specific rabbit immunoglobulin G antibodies (Fig. 2B and C). The lysates of GC-1 cells were immunoprecipitated with an anti-DND1-β antibody followed by immunoblotting with an anti-c-Jun antibody. The result demonstrated that c-Jun was precipitated by DND1-β. When the cell lysates were subjected to immunoprecipitation with an anti-c-Jun antibody, DND1-β was consistently detected in the precipitant. These data suggested that DND1-β interacted with c-Jun in GC-1 cells.

### Dnd1-β co-localizes with c-Jun in GC-1 cells nuclei

**Figure 3:**
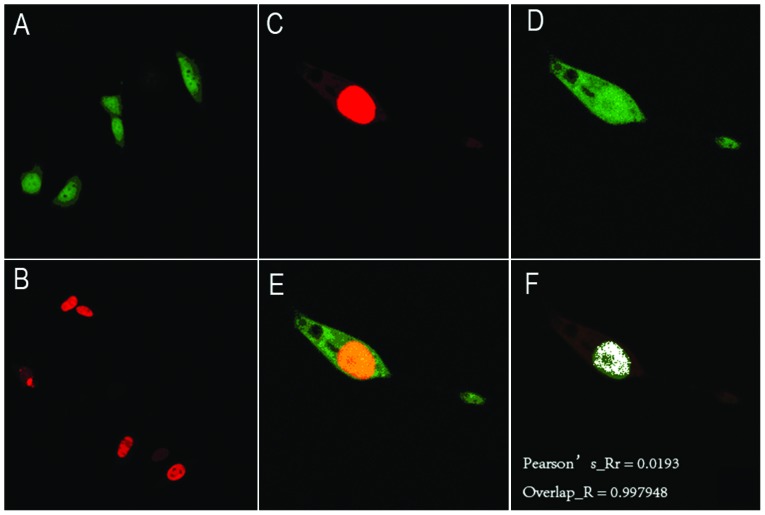
Colocalization of DND1-β and c-Jun in GC-1 spermatogonia cells by confocal microscopy. (A) GC-1 cells were transfected with pEGFP-c3-Dnd1-β plasmid. Green fluorescence was detected in the nuclei of cells. (B) GC-1 cells were transfected with pmRFP-c-Jun plasmid. Red fluorescence was present in the nuclei of cells. (C–E) GC-1 cells were co-transfected with pEGFP-c3-Dnd1-β and pmRFP-c-Jun plasmids. (C) Red fluorescence was found in the nuclei of cells. (D) Green fluorescence was found in the nuclei of cells. (E) C and D were merged and the overlapped areas of the images are shown in yellow. (F) White areas indicate the co-localization of DND1-β and c-Jun with the following correlation coefficients: Pearson’s Rr=0.0193; overlap R=0.997948; DND, dead end, EGFP, enhanced green fluorescent protein; RFP, red fluorescent protein.

As a tight association between DND1-β and c-Jun had been detected in immunoprecipitation and GST pull-down experiments, the next aim was to investigate whether these proteins were present in the same region in cells. Images captured by confocal laser scanning microscopy revealed that GFP-tagged DND1-β and RFP-tagged c-Jun were mainly localized in the nuclei of cells (Fig. 3). Following overlay, yellow co-localized signals were clearly observed (Fig. 3). White areas in Fig. 3F indicate the co-localization between DND1-β and c-Jun with the following correlation coefficients: Pearson’s Rr=0.0193; overlap R=0.997948.

### Interaction of DND1-β and c-Jun stimulates activation of AP-1 transcription

**Figure 4:**
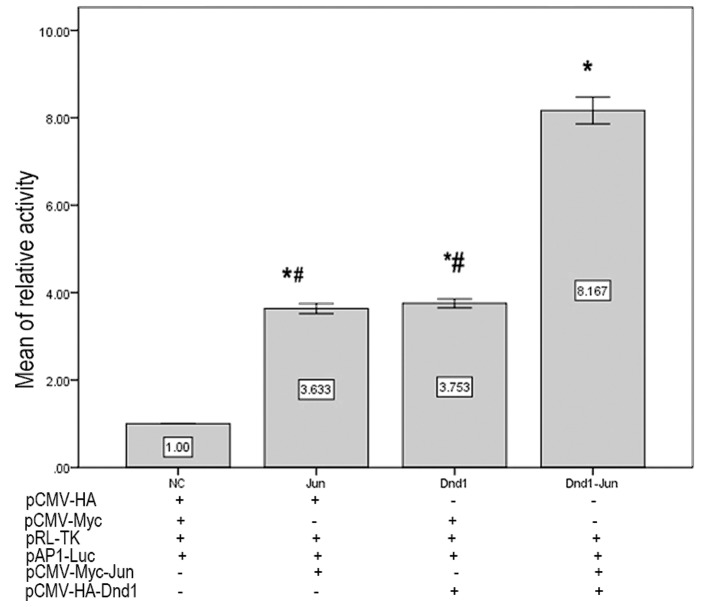
Effects of the interaction between DND1-β and c-Jun on AP-1 transcriptional activity in GC-1 spermatogonia cells. GC-1 cells were transfected with 0.5 ng luciferase reporter AP-1-Luc vector alone (lane 1, NC group) or with 0.3 ng c-Jun expression vector pCMV-Myc-c-Jun (lane 2, c-Jun group) or with 0.3 ng Dnd1-β expression vector pCMV-HA-Dnd1-β (lane 3, Dnd1-β group) or with 0.3 ng pCMV-HA-Dnd1-β and pCMV-Myc-c-Jun (lane 4, co-transfected group). Relative activity of luciferase is presented as the mean ± standard deviation of three independent transfection experiments with each treatment performed in triplicate. ^*^P<0.05 compared with NC; ^#^P<0.05 compared with co-transfected group. DND, dead end; AP, activation protein; CMV, cytomegalovirus, NC, negative control.

To investigate the physiological relevance of the DND1-β-c-Jun interaction, the effect of DND1-β on AP-1 transcriptional activity was analyzed. Either DND1-β or c-Jun alone significantly stimulated luciferase expression (Fig. 4) in GC-1 cells compared with the negative control, but co-transfection with pCMV-Myc-Dnd1-β and pCMV-HA-c-Jun significantly stimulated the luciferase activity further than either individual protein (P<0.05). These results suggested that the interaction between DND1-β and c-Jun activated AP-1 transcription.

## Discussion

DND1 has been reported to be the first protein with an RNA recognition motif (RRM) and has been directly implicated as a heritable cause of spontaneous tumorigenesis (3). To understand in greater detail the mechanism by which DND1-β influences protein expression levels in mice, DND1-β was identified as a c-jun-binding protein by yeast two-hybrid screening. The data from *in vitro* and *in vivo* experiments revealed that DND1-β and c-jun directly interact, and the biological relevance of this interaction was investigated.

AP-1 is a homo- or heterodimeric transcription factor protein composed of other proteins belonging to the Jun, Fos, activating transcription factor and Jun dimerization protein subfamilies (13,14). AP-1 was found at the receiving end of multiple signaling pathways and regulates gene expression in response to a variety of stimuli, including cytokines, growth factors, stress, as well as bacterial and viral infections. AP-1 controls a broad range of biological processes, including proliferation, transformation, cell differentiation, cell migration and apoptosis (15–18). AP-1 activity is induced by multiple environmental insults and physiological stimuli, which activate mitogen-activated protein kinase cascades (19,20). AP-1 activation may result in either induction or prevention of apoptosis, depending on the tissue involved and on the developmental stage of the animal (21–25).

c-Jun is the most extensively investigated AP-1 protein, and is involved in numerous cell functions, including proliferation, apoptosis, survival, tumorigenesis and tissue morphogenesis. The majority of these activities rely on the function of c-Jun as a transcription factor. c-Jun is involved in spermatogenesis, but is also a proto-oncogene. c-Jun is stage-specific with expression levels increasing during active spermatogenesis (26). Following verification of the changes in c-Jun mRNA and protein expression levels at different stages of spermatogenesis in mice, scientists have demonstrated that c-Jun was involved in the transcriptional events regulating the proliferation and differentiation of spermatogenic cells during different stages in the seminiferous epithelium cycle (27–29). c-Jun does not usually act alone; rather, c-Jun physically interacts with other transcription factors, allowing signal integration on promoter DNA for combinatorial transcriptional regulation of gene expression. This type of interaction expands the diversity of c-Jun effects on gene regulation. For instance, interaction with other proteins may promote c-Jun localization to a non-AP-1 site to regulate gene expression.

In the present study, c-Jun was identified as a novel binding partner of DND1-β. *In vivo* binding of DND1-β with c-Jun was identified by immunoprecipitation and immunofluorescence, and *in vitro* interaction between DND1-β and c-Jun was demonstrated by GST pull-down assays.

The dual luciferase reporter assay is a system that employs two luciferase proteins, firefly and renilla, which are assayed independently in one reaction mixture (30,31). AP-1 activity was measured with the dual luciferase reporter gene assay. DND1 or c-Jun alone were found to activate the luciferase reporter to similar extents; however, the two proteins together exerted an effect marginally greater than the sum of the individual effects. This suggested that the interaction between DND1-β and c-Jun subsequently resulted in the activation of AP-1.

In conclusion, DND1-β, which was previously described as an RNA-binding protein and involved in germ cell development, embryonic viability and tumorigenesis, was demonstrated to also interact specifically with the c-Jun transcription factor. The consequence of this interaction is AP-1 activation, which may be important in germ cell development, embryonic viability and tumorigenesis, including that which occurs in TGCT.

## References

[1] Weidinger G, Stebler J, Slanchev K (2003). Dead end, a novel vertebrate germ plasm component, is required for zebrafish primordial germ cell migration and survival. Curr Biol.

[2] Sakurai T, Iguchi T, Moriwaki K, Noguchi M (1995). The ter mutation first causes primordial germ cell deficiency in ter/ter mouse embryos at 8 days of gestation. Dev Growth Differ.

[3] Youngren KK, Coveney D, Peng X (2005). The Ter mutation in the dead end gene causes germ cell loss and testicular germ cell tumours. Nature.

[4] Zhu R, Bhattacharya C, Matin A (2007). The role of dead-end in germ-cell tumor development. Ann N Y Acad Sci.

[5] Cook MS, Munger SC, Nadeau JH, Capel B (2011). Regulation of male germ cell cycle arrest and differentiation by DND1 is modulated by genetic background. Development.

[6] Bhattacharya C, Aggarwal S, Zhu R (2007). The mouse dead-end gene isoform alpha is necessary for germ cell and embryonic viability. Biochem Biophys Res Commun.

[7] Horvay K, Claussen M, Katzer M, Landgrebe J, Pieler T (2006). Xenopus dead end mRNA is a localized maternal determinant that serves a conserved function in germ cell development. Dev Biol.

[8] Peng HQ, Bailey D, Bronson D, Goss PE, Hogg D (1995). Loss of heterozygosity of tumor suppressor genes in testis cancer. Cancer Res.

[9] McIntyre A, Summersgill B, Jafer O (2004). Defining minimum genomic regions of imbalance involved in testicular germ cell tumors of adolescents and adults through genome wide microarray analysis of cDNA clones. Oncogene.

[10] Muller EE, Fayemiwo SA, Lewis DA (2011). Characterization of a novel beta-lactamase-producing plasmid in Neisseria Gonorrhoeae: sequence analysis and molecular typing of host Gonococci. J Antimicrob Chemother.

[11] Tian Y, Ke S, Chen M, Sheng T (2003). Interactions between the aryl hydrocarbon receptor and P-TEFb. Sequential recruitment of transcription factors and differential phosphorylation of C-terminal domain of RNA polymerase II at cyp1a1 promoter. J Biol Chem.

[12] Lauffart B, Howell SJ, Tasch JE, Cowell JK, Still IH (2002). Interaction of the transforming acidic coiled-coil 1 (TACC1) protein with ch-TOG and GAS41/NuBI1 suggests multiple TACC1-containing protein complexes in human cells. Biochem J.

[13] Wagner EF (2001). AP-1 - Introductory remarks. Oncogene.

[14] Shaulian E, Karin M (2001). AP-1 in cell proliferation and survival. Oncogene.

[15] Leppä S, Bohmann D (1999). Diverse functions of JNK signaling and c-Jun in stress response and apoptosis. Oncogene.

[16] Johnson GL, Lapadat R (2002). Mitogen-activated protein kinase pathways mediated by ERK, JNK, and p38 protein kinases. Science.

[17] Shaulian E, Karin M (2002). AP-1 as a regulator of cell life and death. Nat Cell Biol.

[18] Ameyar M, Wisniewska M, Weitzman JB (2003). A role for AP-1 in apoptosis: the case for and against. Biochimie.

[19] Maeda S, Karin M (2003). Oncogene at last - c-Jun promotes liver cancer in mice. Cancer Cell.

[20] Jochum W, Passegué E, Wagner EF (2001). AP-1 in mouse development and tumorigenesis. Oncogene.

[21] Colotta F, Polentarutti N, Sironi M, Mantovani A (1992). Expression and involvement of c-fos and c-jun protooncogenes in programmed cell death induced by growth factor deprivation in lymphoid cell lines. J Biol Chem.

[22] Hilberg F, Aguzzi A, Howells N, Wagner EF (1993). c-jun is essential for normal mouse development and hepatogenesis. Nature.

[23] Smeyne RJ, Vendrell M, Hayward M (1993). Continuous c-fos expression precedes programmed cell death in vivo. Nature.

[24] Estus S, Zaks WJ, Freeman RS, Gruda M, Bravo R, Johnson EM (1994). Altered gene expression in neurons during programmed cell death: identification of c-jun as necessary for neuronal apoptosis. J Cell Biol.

[25] Hafezi F, Steinbach JP, Marti A (1997). The absence of c-fos prevents light-induced apoptotic cell death of photoreceptors in retinal degeneration in vivo. Nat Med.

[26] Cohen DR, Vandermark SE, McGovern JD, Bradley MP (1993). Transcriptional regulation in the testis: a role for transcription factor AP-1 complexes at various stages of spermatogenesis. Oncogene.

[27] Shalini S, Bansal MP (2006). Role of selenium in spermatogenesis: differential expression of cjun and cfos in tubular cells of mice testis. Mol Cell Biochem.

[28] Wolfes H, Kogawa K, Millette CF, Cooper GM (1989). Specific expression of nuclear proto-oncogenes before entry into meiotic prophase of spermatogenesis. Science.

[29] Chieffi P, Angelini F, Pierantoni R (1997). Proto-oncogene activity in the testis of the lizard, Podarcis s. sicula, during the annual reproductive cycle. Gen Comp Endocrinol.

[30] Grentzmann G, Ingram JA, Kelly PJ, Gesteland RF, Atkins JF (1998). A dual-luciferase reporter system for studying recoding signals. RNA.

[31] Lee JY, Kim S, Hwang do W (2008). Development of a dual-luciferase reporter system for in vivo visualization of MicroRNA biogenesis and posttranscriptional regulation. J Nucl Med.

